# Influence of comorbidity of chronic diseases on basic activities of daily living among older adults in China: a propensity score-matched study

**DOI:** 10.3389/fpubh.2024.1292289

**Published:** 2024-04-04

**Authors:** Hongji Zeng, Chen Miao, Rui Wang, Weijia Zhao, Wenjuan Wang, Yahui Liu, Shufan Wei, Anqi Liu, Huibing Jia, Guoxin Li, Junge Zhou, Xuejiao Chen, Qingfeng Tian

**Affiliations:** ^1^School of Public Health, Zhengzhou University, Zhengzhou, China; ^2^Henan Medical College, Zhengzhou, China

**Keywords:** basic activities of daily living, comorbidities of chronic disease, chronic disease, older adults, quality of life

## Abstract

**Rationale:**

With the accelerating process of population aging, the comorbidity of chronic disease (CCD) has become a major public health problem that threatens the health of older adults.

**Objective:**

This study aimed to assess whether CCD is associated with basic activities of daily living (BADL) and explore the factors influencing BADL in older adults.

**Method:**

A cross-sectional community health survey with stratified random sampling among older residents (≥60 years old) was conducted in 2022. A questionnaire was used to collect information on BADL, chronic diseases, and other relevant aspects. Propensity score matching (PSM) was used to match the older adults with and without CCD. Univariate and multivariate logistic regression analyses were used to explore the factors influencing BADL. PSM was used to match participants with single-chronic disease (SCD) and CCD.

**Results:**

Among the 47,720 participants, those with CCD showed a higher prevalence of BADL disability (13.07%) than those with no CCD (6.33%) and SCD (7.39%). After adjusting for potential confounders with PSM, 6,513 pairs of cases with and without CCD were matched. The univariate analysis found that the older adults with CCD had a significantly higher prevalence of BADL disability (13.07%, 851 of 6,513) than those without CCD (9.83%, 640 of 6,513, *P* < 0.05). The multivariate logistic regression analysis revealed that CCD was a risk factor for BADL in older adults [OR = 1.496, 95% CI: 1.393–1.750, *P* < 0.001]. In addition, age, educational level, alcohol intake, social interaction, annual physical examination, retirement benefits, depression, weekly amount of exercise, and years of exercise were related to BADL disability (*P* < 0.05). PSM matching was performed on participants with CCD and SCD and showed that the older adults with CCD had a significantly higher prevalence of BADL disability (13.07%, 851 of 6,513) than those with SCD (11.39%, 742 of 6,513, *P* < 0.05).

**Conclusion:**

The older adults with CCD are at a higher risk of BADL disability than their counterparts with no CCD or SCD. Therefore, we advocate paying attention to and taking measures to improve the health and quality of life of these individuals.

## 1 Introduction

The challenge of an aging population is a relatively recent phenomenon in historical terms, but its acceleration has become increasingly apparent since the beginning of the 21st century (1). As of 2020, the total number of Chinese adults aged ≥60 years has reached 264.02 million, accounting for 18.70% of the total population (2). Research indicates that China will enter a highly aging society in 2035, with the proportion of older individuals exceeding 20% (3).

With the global population aging, there is a growing emphasis on chronic diseases due to the increasing life expectancy (4). The World Health Organization (WHO) reports that chronic diseases account for 38 million deaths annually (5). In the United States, half of all adults are affected by chronic diseases (6). Similarly, in Europe, a study found that over one-third of European adults suffer from chronic diseases, which contribute to more than 80% of total deaths (7). In China, over 300 million individuals have been diagnosed with chronic diseases, representing 86.8% of all-cause mortality each year. Additionally, chronic diseases have become the primary cause of disability among Chinese adults (8).

According to the WHO, comorbidity of chronic diseases (CCD) refers to the presence of two or more chronic diseases in a single individual (9). As chronic diseases continue to be prevalent, CCD has emerged as a significant global public health concern (10). The complex causes of chronic diseases, including the correlation with various behavioral factors and the existence of common risk factors shared among different diseases, contribute to CCD (11). Previous studies have shown that 38% of American adults have experienced CCD (12), while around three-quarters of European Union citizens aged 65 and above have been affected (13). With the rapid aging of the population, CCD is expected to pose significant challenges and threats to healthcare systems (10).

BADL refers to the basic daily movements that individuals perform to maintain their independence, such as dressing and grooming (14). It is a crucial indicator of physical and cognitive ability in older adults and is considered a vital component of healthy aging and independent living (15). Therefore, a comprehensive understanding of BADL is essential for future health-service planning. However, most studies of BADL in the older adults worldwide are based on a single chronic disease. There is insufficient research on the association between CCD and BADL. Therefore, the current study was carried out to analyze the impact of CCD on BADL in older adults to provide references for improving their quality of life.

## 2 Method

### 2.1 Procedures and study participants

In 2021, we conducted a preliminary survey in Zhongmou County, Henan Province, to estimate the sample size required for the formal survey. The preliminary survey lasted 1 month with the same selection criteria as the formal study. During this period, 3,245 participants were included, and the prevalence of CCD was 15.79%. Additionally, we conducted reliability and validity tests based on the preliminary survey because some of the assessments may be affected by subjective factors. The results showed Cronbach's α of 0.932 and a KMO of 0.969. Based on the CCD prevalence of 15.79%, we estimated the sample size, assuming that the probability of type I error was α = 0.05, *P* = 15.79, and the margin of error was 0.05. The formula was as follows:


$$\begin{array}{l} n\;=\;{\left(Z_{\alpha /2}\right)}^{2}\times \;\left[p\;\left(1-p\right)\right]/\;\left(d^{2}\right)\approx 20,432 \end{array}$$

Based on the expected data loss rate of 20%, we estimated that at least 24,518 participants were required.

In 2022, we conducted a community health survey using a stratified random sampling method among the older adults (aged ≥60 years) from 635 communities in county-level administrative regions under the jurisdiction of Zhengzhou City, Henan Province, including Zhongyuan, Erqi, Guancheng, Jinshui, Shangjie, Huiji, Gongyi, Xingyang, Xinmi, Dengfeng, and Zhongmou. This study was approved by the Ethics Committee of Zhengzhou University (Ethical Number: ZZUIRB2022-07) and conducted in accordance with the relevant guidelines and local regulations. Written informed consent was obtained from each patient before enrollment in the study.

During the survey, we conducted a dynamic data entry for CCD prevalence to monitor the sample saturation rate throughout the study. Sample saturation refers to the point at which the sample size reaches a certain level, and there is no significant change in CCD prevalence with further increases in the sample size. We found that the sample began to saturate when the sample size reached 45,289. We then stopped the survey after 2 weeks.

**Figure 1:**
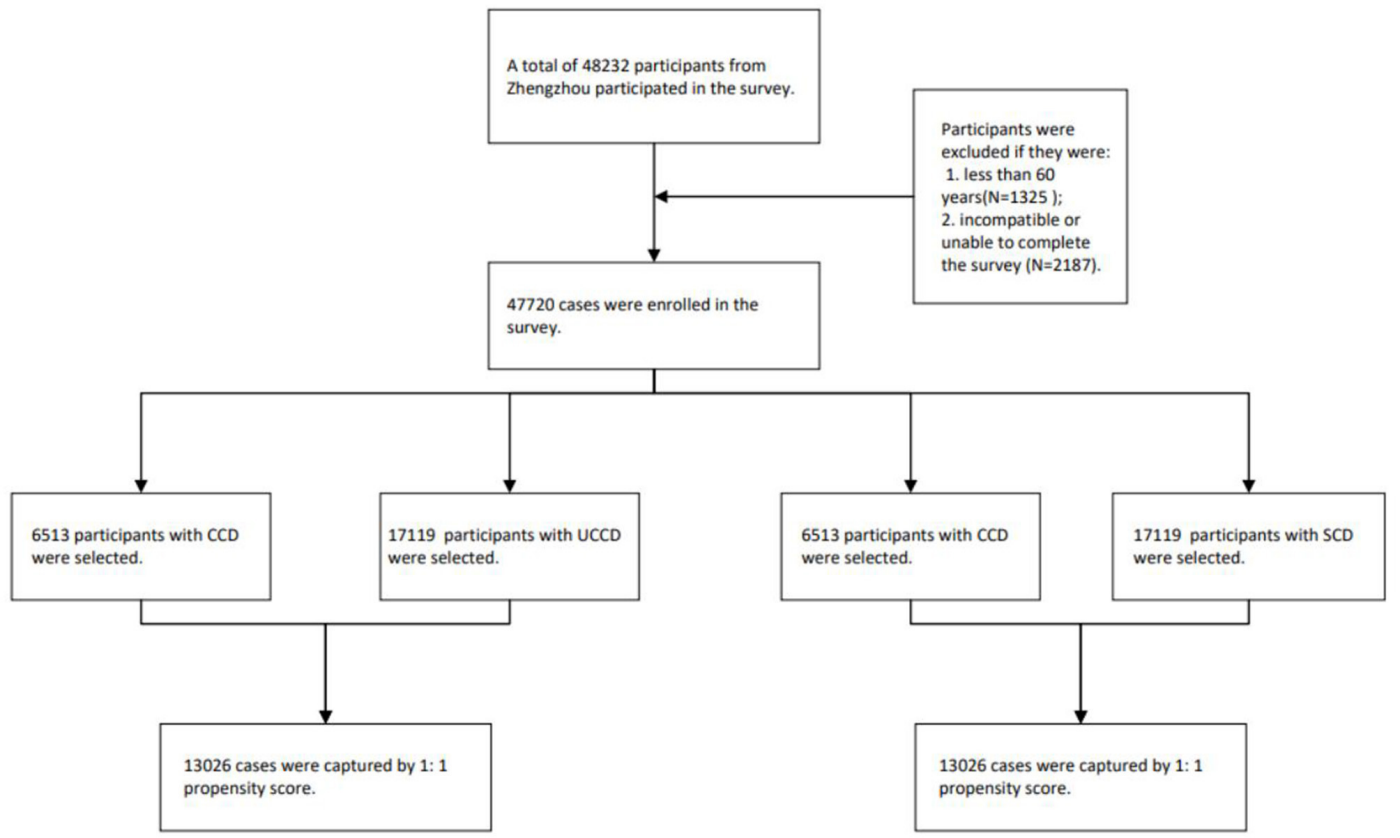
The flowchart of participant selection of this study.

The invited participants were informed of the topic before their tentative consent to complete the survey. Participants were excluded if they met the following criteria: (1) < 60 years of age and (2) unable to cooperate or complete the survey due to aphasia, severe psychological disorders, or cognitive impairments. Finally, 48,232 participants were enrolled. After excluding 512 incomplete cases, 47,720 cases were included in this study. PSM was used to eliminate the endogeneity of control variables and the interference of confounding factors and to improve the accuracy of the results. In the first PSM, 6,513 pairs of cases with CCD and no CCD were matched, while in the second PSM, 6,513 pairs of cases with CCD and SCD were matched. None of the variables used in the final analysis contained missing data. The flowchart is shown in Figure 1.

### 2.2 Assessments

This study used a self-designed questionnaire, which has been reviewed and approved by experts at Zhengzhou University. All components of the questionnaire were derived from standardized sub-questionnaires.

#### 2.2.1 Explained variable

BADL is the explained variable, which was assessed using the modified Barthel Index (MBI). The scores range from 0 to 100, with higher scores indicating greater BADL. The scale covers 10 subscales, namely, fecal continence, micturition continence, eating, dressing, bathing, personal hygiene, toileting, moving, walking, and climbing stairs (16). It was reported that when the score was above 60, most older adults could manage their daily lives independently, while the older adults with an MBI < 60 could not independently complete many daily activities (17). As a result, participants were divided into two groups according to whether or not their MBI scores were higher than 60. Cronbach's α for the MBI was 0.916. The assessment of BADL was based on a face-to-face investigation (Appendix A).

#### 2.2.2 Explanatory variable

The condition of chronic disease was the explanatory variable. CCD refers to an individual simultaneously suffering from two or more chronic diseases. No CCD refers to an individual suffering from < 1 chronic disease. SCD refers to an individual with a chronic disease. First, the participants were divided into CCD and no CCD groups for the first PSM. For further analysis, the participants were divided into the CCD and SCD groups for the second PSM. In this study, 16 chronic diseases, namely hypertension, diabetes, heart disease, stroke and cerebrovascular disease, emphysema, asthma or pneumonia, prostate disease, gastrointestinal ulcer, Parkinson's disease, dementia, epilepsy, dyslipidemia, cholecystitis or gallstone, chronic nephritis, hysteromyoma, and hepatitis, were included. Throughout the study, we requested primary healthcare institutions to provide patients' medical records to ensure the accuracy of the disease status documentation (18).

#### 2.2.3 Control variables

Control variables included (1) sociodemographic characteristics: age (years), sex (male/female), type of residence (urban/rural), body mass index (BMI, body weight divided by squared of height, kg/m^2^), educational level (under junior high school/junior high school or above), and ethnicity (Han/minority nationality); (2) social support: cohabitation (living alone/living with others), daily care from offspring (more than once per week), and social interaction (more than once per week); (3) health behavior: smoking (one cigarette at least a day for at least 6 months), alcohol intake (at least once a week for at least 6 months), balanced diet, weekly amount of exercise (min), years of exercise (year), and annual physical examination (at least for 3 years); (4) economic status: retirement benefits; and (5) mental health: depression. The specific assignments are shown in Appendix B.

The assessment of a balanced diet was subjective, and we explained to each participant that a diet high in oil and salt or imbalanced in meat and vegetables was considered unbalanced. According to relevant definitions, exercise was considered a heart rate higher than 50% ^*^ (220–age). However, it was not always possible to completely follow this point. We generally adopted conventional practices and believed that brisk walking or higher-intensity activities were qualified as exercises (19, 20). Finally, we used the Geriatric Depression Scale (GDS) to assess mental health and considered scores above 9 points to indicate the presence of depression (18). The assessment of control variables was based on ID information or self-reports.

### 2.3 Statistical methods

The counting data were expressed as the number of cases and percentage (*n*, %), and the comparison between groups was conducted using a chi-square test if applicable. After passing the tests for normality and homogeneity of variance, the measurement data were expressed as mean (SD), and a *t*-test was performed between the groups. The Mann–Whitney *U*-test was used when the data were skewed and the variance was homogeneous. The values were expressed as median (interquartile range). PSM can eliminate the endogeneity and confounding factors of control variables to improve the accuracy of the results. A probit regression model was used to estimate the propensity scores for CCD vs. no CCD and SCD vs. CCD participants. After matching, univariate and multivariate logistic regression analyses were performed to test factors influencing BADL. The second PSM was performed with the same setting between the CCD and SCD groups to test the influence of CCD on BADL compared to SCD. The formula for prevalence was the number of individuals with the disease (CCD or SCD) divided by the total number of individuals in the group. SPSS 21.0 was used for statistical analysis. Differences were considered statistically significant if *P*-values were < 0.05.

## 3 Result

### 3.1 Characteristics of participants

A total of 47,720 participants were enrolled in the study (13.65% CCD, 86.35% no CCD, and 35.87% SCD). The characteristics of the participants are shown in Table 1.

**Table 1:** Characteristics of participants.

**Item**				**Result**	**Cases**	**Percentage (%)**
Sex				Male	24,710	51.78
				Female	23,010	48.22
Type of residence				Urban	32,059	67.18
				Rural	15,661	32.82
Ethnicity				Han	47,345	99.21
				Minority nationality	375	0.79
Educational level				Under junior high school	17,902	37.51
				Junior high school or above	29,818	62.49
BMI				18.5–23.9 kg/m^2^	35,868	75.16
				< 18.5 kg/m^2^	3,335	6.99
				>23.9 kg/m^2^	8,517	17.85
Cohabitation				Living alone	12,492	26.18
				Living with others	35,228	73.82
Balanced diet				Yes	38,976	81.68
				No	8,744	18.32
Smoking				No	43,651	91.47
				Yes	4,069	8.53
Alcohol intake				No	44,591	93.44
				Yes	3,129	6.56
Social interaction				Yes	47,036	98.57
				No	684	1.43
Daily care from offspring				Yes	47,597	99.74
				No	123	0.26
Annual physical examination				Yes	46,808	98.09
				No	912	1.91
Retirement benefits				Yes	47,504	99.55
				No	216	0.45
Depression				Not suffering	47,474	99.48
				Suffering	246	0.52
Condition of CCD				CCD	41,207	86.35
				No CCD	6,513	13.65
BADL				BADL no disability	44,261	92.75
				BALD disability	3,459	7.25
Chronic diseases				Suffering	23,632	49.52
				Not suffering	24,088	50.48
Condition of SCD				SCD	17,119	35.87
				Not SCD	30,601	64.13
Total					47,720	100.0
**Item**	**Minimum**	**Maximum**	**Average**	**Standard deviation**	**Median**	
Age (years)	63.00	95.00	72.28	6.17	71.00	
Weekly amount of exercise (min)	0.00	900.00	15.47	25.34	0.00	
Years of exercise (years)	0.00	50.00	2.38	5.02	0.00	

### 3.2 BADL of the older adults with different conditions of chronic diseases

A total of 3,459 (7.25%) participants expressed BADL disabilities. The prevalence of BADL disability in the older adults with CCD (13.07%) was significantly higher (*P* < 0.05) than that in their counterparts without CCD (6.33%). Of the 23,632 elderlies with chronic diseases, 2,116 cases (8.95%) expressed BADL disability. The older adults with CCD (13.07%) showed a significantly higher (*P* < 0.05) prevalence of BADL disability than those with SCD (7.39%).

### 3.3 PSM results for cases with and without CCD

A total of 6,513 participants (13.64%) had CCD. Before PSM, the age, sex, cohabitation, BMI, educational level, ethnicity, social situation interaction, smoking, alcohol intake, balanced diet, weekly amount of exercise, years of exercise, annual physical examination, pension, and retirement benefits between participants with and without CCD showed significant differences (*P* < 0.05). After PSM matching, 6,513 pairs of cases with and without CCD were matched. There were no significant differences (*P* > 0.05) in the above variables between the two matched groups, as shown in Table 2 and Appendix C.

**Table 2:** Comparison of control variables between cases with CCD and no CCD before and after PSM.

**Item**	**Before PSM**				**After PSM**			
	**Participants with CCD (*****n*** = **6,513)**	**Participants with no CCD (*****n*** = **41,207)**	*t*/*U*/*X*^2^	***P***	**Participants with CCD (*****n*** = **6,513)**	**Participants with no CCD (*****n*** = **6,513)**	***t/U/X** ^2^*	***P***
**Sex (%)**								
Male	1,790 (27.48)	22,920 (55.62)	1,783.419	< 0.001^*^	1,790 (27.48)	1,821 (27.96)	0.368	0.544
Female	4,723 (72.52)	18,287 (44.38)			4,723 (72.52)	4,692 (72.04)		
**Type of residence (%)**								
Urban	4,326 (66.42)	27,733 (67.30)	1.978	0.160	4,326 (66.42)	4,256 (65.34)	1.674	0.196
Rural	2,187 (33.58)	13,474 (32.70)			2,187 (33.58)	2,257 (34.65)		
**Ethnicity (%)**								
Han	6,444 (98.94)	40,901 (99.26)	7.241	0.007^*^	6,444 (98.94)	6,447 (98.99)	0.067	0.795
Minority nationality	69 (1.06)	306 (0.74)			69 (1.06)	66 (1.01)		
**Educational level (%)**								
Under junior high school	3,289 (50.50)	14,613 (35.46)	542.464	< 0.001^*^	3,289 (50.50)	3,357 (51.54)	1.421	0.233
Junior high school or above	3,224 (49.50)	26,594 (64.54)			3,224 (49.50)	3,156 (48.46)		
**BMI (%)**								
18.5–23.9 kg/m^2^	3,783 (58.08)	32,085 (77.86)	1,187.043	< 0.001^*^	3,783 (58.08)	3,689 (56.64)	2.813	0.245
< 18.5 kg/m^2^	817 (12.54)	2,518 (6.11)			817 (12.54)	852 (13.08)		
>23.9 kg/m^2^	1,913 (29.38)	6,604 (16.03)			1,913 (29.38)	1,972 (30.28)		
**Cohabitation (%)**								
Living alone	4,915 (75.46)	30,313 (73.56)	10.525	0.001^*^	4,915 (75.46)	4,936 (75.79)	0.184	0.668
Living with others	1,598 (24.54)	10,894 (26.44)			1,598 (24.54)	1,577 (24.21)		
**Balanced diet (%)**								
Yes	5,122 (78.64)	33,854 (82.16)	46.383	< 0.001^*^	5,122 (78.64)	5,143 (78.97)	0.203	0.653
No	1,391 (21.36)	7,353 (17.84)			1,391 (21.36)	1,370 (21.03)		
**Smoking (%)**								
No	6,234 (95.72)	37,417 (90.80)	174.097	< 0.001^*^	6,234	6,218 (95.47)	0.467	0.494
					(95.72)			
Yes	279 (4.28)	3,790 (0.92)			279 (4.28)	295 (4.53)		
**Alcohol intake (%)**								
No	6,276 (96.36)	38,315 (92.98)	104.825	< 0.001^*^	6,276 (96.36)	6,276 (96.36)	0	1.000
Yes	237 (3.64)	2,892 (7.02)			237 (3.64)	237 (3.64)		
**Social interaction (%)**								
Yes	6,367 (97.76)	40,669 (98.69)	34.880	< 0.001^*^	6,367 (97.76)	6,370 (97.80)	0.032	0.858
No	146 (2.24)	538 (1.31)			146 (2.24)	143 (2.20)		
**Daily care from offspring (%)**								
Yes	6,489 (99.63)	41,108 (99.76)	3.598	0.058	6,489 (99.63)	6,495 (99.72)	0.860	0.354
No	24 (0.37)	99 (0.24)			24 (0.37)	18 (0.28)		
**Annual physical examination (%)**								
Yes	6,342 (97.37)	40,466 (98.20)	20.533	< 0.001^*^	6,342 (97.37)	6,363 (97.70)	1.409	0.235
No	171 (2.63)	741 (1.80)			171 (2.63)	150 (2.30)		
**Retirement benefits (%)**								
Yes	6,467 (99.29)	41,037 (99.59)	10.769	< 0.001^*^	6,467 (99.29)	6,476 (99.43)	0.982	0.322
No	46 (0.71)	170 (0.41)			46 (0.71)	37 (0.57)		
**Depression (%)**								
Suffering	48 (0.73)	198 (0.48)	7.214	0.007^*^	48 (0.73)	42 (0.64)	0.403	0.526
Not suffering	6,465 (99.27)	41,009 (99.52)			6,465 (99.27)	6,471 (99.36)		
Age [years, median (P_25_, P_75_)]	72.00 (68.00, 76.00)	71.00 (67.00, 76.00)	10.322	< 0.001^*^	72.00 (68.00, 76.00)	72.00 (68.00, 77.00)	0.247	0.805
Weekly amount of exercise [min, median (P_25_, P_75_)]	0.00 (0.00, 40.00)	0.00 (0.00, 30.00)	7.491	< 0.001^*^	0.00 (0.00, 40.00)	0.00 (0.00, 40.00)	1.926	0.054
Years of exercise [years, median (P_25_, P_75_)]	0.00 (0.00, 3.00)	0.00 (0.00, 3.00)	3.973	< 0.001^*^	0.00 (0.00, 3.00)	0.00 (0.00, 3.00)	1.826	0.068

ATT (average treatment effect on the treated) = −0.33; Std. Error: 0.06; *t* = 6.020; *P* < 0.001.

^*^P < 0.05.

*t*/*U*/*X*^2^, eigenvalue; *t*, test; *U*, Mann–Whitney U-test; *X*^2^, chi-square test.

### 3.4 Univariate analysis of BADL in participants

After PSM, among a total of 13,026 participants, 11,535 (88.55%) expressed BADL no disability and 1,491 (11.45%) BADL disability. Participants with CCD showed a significantly higher prevalence of BADL disability than those with no CCD (13.07% vs. 9.83%, 1.330 times, *P* < 0.001). There was a statistically significant difference in age, cohabitation, type of residence, educational level, ethnicity, daily care from offspring, social interaction, alcohol intake, balanced diet, weekly amount of exercise, years of exercise, annual physical examination, retirement benefits, and depression (*P* < 0.05), as shown in Tables 3.1, 3.2.

**Table 3.1:** Univariate analysis of the BADL in participants after PSM (%, counting data).

**Item**	**Result**	**BADL disability (*n* = 1,491)**	**BADL no disability (*n* = 11,535)**	**Total**	**χ^2^**	***P***
Sex (%)	Female	1,086 (72.84)	8,329 (72.21)	9,415 (72.28)	0.262	0.609
	Male	405 (27.16)	3,206 (27.79)	3,611 (27.72)		
Total		1,491	11,535	13,026		
Type of residence (%)	Rural	506 (33.94)	3,938 (34.14)	4,444 (34.12)	0.024	0.877
	Urban	985 (66.06)	7,597 (65.86)	8,582 (65.88)		
Total		1,491	11,535	13,026		
BMI (%)	18.5–23.9 kg/m^2^	822 (55.13)	6,650 (57.65)	7,472	4.637	0.098
	< 18.5 kg/m^2^	213 (14.29)	1,456 (12.62)	1,669		
	>23.9 kg/m^2^	456 (30.58)	3,429 (29.73)	3,885		
Total		1,491	11,535	13,026		
Ethnicity (%)	Minority nationality	23 (1.54)	112 (0.97)	135 (1.04)	4.206	0.040^*^
	Han	1,468 (98.46)	11,423 (99.03)	12,891 (98.96)		
Total		1,491	11,535	13,026		
Educational level (%)	Junior high school or above	425 (28.50)	5,955 (51.63)	6,380 (48.98)	282.451	< 0.00 ^*^
	Under junior high school	1,066 (71.50)	5,580 (48.37)	6,646 (51.02)		
Total		1,491	11,535	13,026		
Cohabitation (%)	Living with others	1,054 (70.69)	8,797 (76.26)	9,851 (75.63)	22.244	< 0.001^*^
	Living alone	437 (29.31)	2,738 (23.74)	3,175 (24.37)		
Total		1,491	11,535	13,026		
Balanced diet (%)	No	421 (28.24)	2,340 (20.29)	2,761 (21.20)	49.959	< 0.001^*^
	Yes	1,070 (71.76)	9,195 (79.71)	10,265 (78.80)		
Total		1,491	11,535	13,026		
Smoking (%)	No	1,413 (94.77)	11,039 (95.70)	12,452 (95.59)	2.719	0.099
	Yes	78 (5.23)	496 (4.30)	574 (4.41)		
Total		1,491	11,535	13,026		
Alcohol intake (%)	No	1,415 (94.90)	11,137 (96.55)	12,552 (96.36)	10.213	0.001^*^
	Yes	76 (5.10)	398 (3.45)	320 (2.46)		
Total		1,491	11,535	13,026		
Social interaction (%)	No	131 (8.79)	158 (1.37)	289 (2.22)	334.747	< 0.001^*^
	Yes	1,360 (91.21)	11,377 (98.63)	12,737 (97.78)		
Total		1,491	11,535	13,026		
Daily care from offspring (%)	No	9 (0.60)	33 (0.29)	42 (0.32)	4.142	0.042^*^
	Yes	1,482 (99.40)	11,502 (99.71)	12,984 (99.68)		
Total		1,491	11,535	13,026		
Annual physical examination (%)	No	86 (5.77)	235 (2.04)	321 (2.46)	76.454	< 0.001^*^
	Yes	1,405 (94.23)	11,300 (97.96)	12,705 (97.54)		
Total		1,491	11,535	13,026		
Retirement benefits (%)	No	35 (2.35)	48 (0.42)	83 (0.64)	77.784	< 0.001^*^
	Yes	1,456 (97.65)	11,487 (99.58)	12,943 (99.36)		
Total		1,491	11,535	13,026		
Depression (%)	No	1,449 (97.18)	11,487 (99.58)	12,936 (99.31)	110.909	< 0.001^*^
	Yes	42 (2.82)	48 (0.42)	90 (0.69)		
Total		1,491	11,535	13,026		
Condition of CCD (%)	No CCD	640 (9.83)	5,873 (90.17)	6,513 (50.00)	33.719	< 0.001^*^
	CCD	851 (13.06)	5,662 (86.94)	6,513 (50.00)		
Total		1,491	11,535	13,026		

^*^P < 0.05.

**Table 3.2:** Univariate analysis of the BADL in participants after PSM ($\bar{x}$ ± *s*, measurement data).

**Item**	**BADL disability (*n* = 1,491)**	**BADL no disability (*n* = 11,535)**	***U***	***P***
Age [years, median (P_25_, P_75_)]	76.00 (71.00, 82.00)	71.00 (68.00, 76.00)	22.087	< 0.001^*^
Weekly amount of exercise [min, median (P_25_, P_75_)]	0.00 (0.00, 0.00)	0.00 (0.00, 45.00)	14.042	< 0.001^*^
Years of exercise [years, median (P_25_, P_75_)]	0.00 (0.00, 0.00)	0.00 (0.00, 3.00)	16.540	< 0.001^*^

^*^P < 0.05.

### 3.5 Multivariate logistic regression analysis of BADL disability

A multivariate logistic regression analysis was carried out with BADL as the dependent variable and variables showing statistical significance in the univariate analysis as the independent variables. The results showed that CCD was a risk factor for BADL in older adults (OR = 1.496, 95% CI: 1.393–1.750). Moreover, age (OR = 1.102, 95% CI: 1.021–1.316), educational level (OR = 2.253, 95% CI: 1.290–2.705), weekly amount of exercise (OR = 0.996, 95% CI: 0.989–0.998), years of exercise (OR = 0.836, 95% CI: 0.652–0.941), alcohol intake (OR = 2.242, 95% CI: 1.939–2.688), social interaction (OR = 0.136, 95% CI: 0.058–0.464), annual physical examination (OR = 0.480, 95% CI: 0.152–0.772), retirement benefits (OR = 0.319, 95% CI: 0.196–0.669) and depression (OR = 0.124, 95% CI: 0.049–0.726) were influencing factors of BADL in older adults (*P* < 0.05), as shown in Table 4.

**Table 4:** Multivariate logistic regression analysis of BADL of older adults.

**Item**		**β**	**Standard deviation**	***z***	**Wald χ^2^**	***P***	**OR**	**95% CI**
CCD	No CCD	1.00 (ref.)						
	CCD	0.404	0.060	6.754	45.610	< 0.001^*^	1.498	1.393–1.750
Educational level	Junior high school or above	1.00 (ref.)						
	Under junior high school	0.813	0.066	12.377	153.182	< 0.001^*^	2.253	1.290–2.705
Alcohol intake	No	1.00 (ref.)						
	Yes	0.807	0.140	5.744	32.999	< 0.001^*^	2.242	1.939–2.688
Social interaction	No	1.00 (ref.)						
	Yes	−1.989	0.137	−14.470	209.382	< 0.001^*^	0.136	0.058–0.464
Annual physical examination	No	1.00 (ref.)						
	Yes	−0.732	0.147	−4.976	24.757	< 0.001^*^	0.480	0.152–0.772
Retirement benefits	No	1.00 (ref.)						
	Yes	−1.141	0.256	−4.462	19.905	< 0.001^*^	0.319	0.196–0.669
Depression	Suffering	1.00 (ref.)						
	Not suffering	−2.081	0.236	−8.826	77.903	< 0.001^*^	0.124	0.049–0.726
Daily care from offspring	No	1.00 (ref.)						
	Yes	−0.165	0.450	−0.365	0.133	0.715	0.848	0.688–1.050
Cohabitation	Living alone	1.00 (ref.)						
	Living with others	−0.094	0.070	−1.344	1.806	0.179	0.910	0.858–1.126
Ethnicity	Han	1.00 (ref.)						
	Minority nationality	−0.439	0.266	−1.649	2.718	0.099	0.644	0.082–1.013
Balanced diet	No	1.00 (ref.)						
	Yes	0.003	0.069	0.039	0.002	0.969	1.003	0.871–1.142
Age		1.00 (ref.)						
		0.097	0.005	21.031	442.295	< 0.001^*^	1.102	1.021–1.316
Weekly amount of exercise		1.00 (ref.)						
		−0.004	0.001	−2.693	7.252	0.007^*^	0.996	0.989–0.998
Years of exercise		1.00 (ref.)						
		−0.179	0.019	−9.336	87.168	< 0.001^*^	0.836	0.652–0.941

^*^P < 0.05.

BADL disability = 1, BADL no disability = 0.

### 3.6 PSM of participants with CCD and with SCD

A total of 23,632 participants with chronic diseases were further analyzed using PSM. Before PSM, there were significant differences in sex, educational level, balanced diet, smoking, alcohol intake, social interaction, daily care from offspring, annual physical examination, age, BMI, and weekly amount of exercise (*P* < 0.05). After PSM, there were 6,513 pairs of cases with CCD and SCD while no significant differences were observed in the above variables (*P* > 0.05), as shown in Table 5 and Appendix D. Furthermore, the prevalence of BADL disability in the older adults with CCD was significantly higher than that in the older adults with SCD (13.07% vs. 11.39%, 1.179 times, *P* < 0.05).

**Table 5:** Comparison of the general information of the group with CCD and with SCD before and after PSM.

**Item**	**Before PSM**				**After PSM**			
	**Participants with CCD (*****n*** = **6,513)**	**Participants with SCD (*****n*** = **17,119)**	***t/U/X** ^2^*	***P***	**Participants with CCD (*****n*** = **6,513)**	**Participants with SCD (*****n*** = **6,513)**	***t/U/X** ^2^*	***P***
**Sex (%)**								
Male	1,790 (27.48)	7,421 (43.35)	499.336	< 0.001^*^	1,790 (27.48)	1,813 (27.84)	0.203	0.652
Female	4,723 (72.52)	9,698 (56.65)			4,723 (72.52)	4,700 (72.16)		
**Type of residence (%)**								
Urban	4,326 (66.42)	11,216 (65.51)	1.709	0.191	4,326 (66.42)	4,235 (65.02)	2.822	0.091
Rural	2,187 (33.58)	5,903 (34.49)			2,187 (33.58)	2,278 (34.98)		
**Ethnicity (%)**								
Han	6,444 (98.94)	16,982 (99.20)	3.667	0.056	6,444 (98.94)	6,457 (99.14)	1.365	0.243
Minority nationality	69 (1.06)	137 (0.80)			69 (1.06)	56 (0.86)		
**Educational level (%)**								
Under junior high school	3,289 (50.50)	7,338 (42.86)	111.118	< 0.001^*^	3,289 (50.50)	3,230 (49.59)	1.069	0.301
Junior high school or above (%)	3,224 (49.50)	9,781 (57.14)			3,224 (49.50)	3,283 (50.41)		
**BMI (%)**								
18.5–23.9 kg/m^2^	3,783 (58.08)	9,485 (55.41)	31.557	< 0.001^*^	3,783 (58.08)	3,684 (56.56)	3.417	0.181
< 18.5 kg/m^2^	817 (12.54)	2,628 (15.35)			817 (12.54)	867 (13.31)		
>23.9 kg/m^2^	1,913 (29.38)	5,006 (29.24)			1,913 (29.38)	1,962 (30.12)		
**Cohabitation (%)**								
Living alone	4,915 (75.46)	12,784 (74.68)	1.555	0.212	4,915 (75.46)	4,990 (76.62)	2.370	0.124
Living with others	1,598 (24.54)	4,335 (25.32)			1,598 (24.54)	1,523 (23.38)		
**Balanced diet (%)**								
Yes	5,122 (78.64)	13,734 (80.23)	0.212	< 0.001^*^	5,122 (78.64)	5,103 (78.35)	0.164	0.685
No	1,391 (21.36)	3,385 (19.77)			1,391 (21.36)	1,410 (21.65)		
**Smoking (%)**								
No	6,234 (95.72)	15,925 (93.03)	58.455	< 0.001^*^	6,234 (95.72)	6,208 (95.32)	1.212	0.271
Yes	279 (4.29)	1,194 (6.97)			279 (4.29)	305 (4.68)		
**Alcohol intake (%)**								
No	6,276 (96.36)	16,119 (94.16)	46.143	< 0.001^*^	6,276 (96.36)	6,261 (96.13)	0.478	0.489
Yes	237 (3.64)	1,000 (5.84)			237 (3.64)	252 (3.87)		
**Social interaction (%)**								
Yes	6,367 (97.76)	16,816 (98.23)	5.632	0.018^*^	6,367 (97.76)	6,374 (97.87)	0.176	0.675
No	146 (2.24)	303 (1.77)			146 (2.24)	139 (2.13)		
**Daily care from offspring (%)**								
Yes	6,489 (99.63)	17,083 (99.79)	4.663	0.031^*^	6,489 (99.63)	6,493 (99.69)	0.365	0.546
No	24 (0.37)	36 (0.21)			24 (0.37)	20 (0.31)		
**Annual physical examination (%)**								
Yes	6,342 (97.37)	16,783 (98.04)	9.872	< 0.001^*^	6,342 (97.37)	6,347 (97.45)	0.076	0.783
No	171 (2.63)	336 (1.96)			171 (2.63)	166 (2.55)		
**Retirement benefits (%)**								
Yes	6,467 (99.29)	17,024 (99.45)	1.822	0.177	6,467 (99.29)	6,467 (99.29)	0.000	1.000
No	46 (0.71)	95 (0.55)			46 (0.71)	46 (0.71)		
**Depression (%)**								
Suffering	48 (0.73)	198 (0.48)	7.214	0.007^*^	48 (0.73)	40 (0.12)	0.732	0.392
Not suffering	6,465 (99.27)	41,009 (99.52)			6,465 (99.27)	6,473 (99.88)		
Age [years, median (P_25_, P_75_)]	71.00 (68.00, 76.00)	72.00 (68.00, 76.00)	4.955	< 0.001^*^	72.00 (68.00, 76.00)	72.00 (68.00, 76.00)	1.466	0.143
Weekly amount of exercise [min, median (P_25_, P_75_)]	0.00 (0.00, 40.00)	0.00 (0.00, 40.00)	3.158	0.002^*^	0.00 (0.00, 40.00)	0.00 (0.00, 40.00)	1.858	0.063
Years of exercise [years, median (P_25_, P_75_)]	0.00 (0.00, 3.00)	0.00 (0.00, 3.00)	0.925	0.355	0.00 (0.00, 3.00)	0.00 (0.00, 3.00)	1.501	0.133

ATT = −0.042; Std. Error = 0.005; *t* = −7.701; *P* < 0.001.

^*^ *P* < 0.05.

## 4 Discussion

### 4.1 The prevalence of BADL disability in older adults

The prevalence of BADL disability among the older adults ranged from 6% to 40% (14, 15, 17, 21, 22). These differences may be attributed to inconsistent evaluation criteria and economic inequality across regions (23). In the current study, the prevalence of BADL disability was 7.25%, which was similar to 8.78% reported by Cui et al. (21) and 6% by Zhang et al. (14) and slightly lower than 13% obtained by Connolly et al. (22) using data from the Irish longitudinal study on aging. This may be due to different definitions of older individuals. In this study, the participants were 60 years and older, whereas Connolly's study focused on individuals aged >65 years. According to a previous study (24), age has been identified as an influencing factor of BADL among older adults, which aligns with the present study. Tissues and organs, including the brain, tend to exhibit signs of aging as a person grows older. Age-related changes may manifest as a decline in the quantity and quality of information processing, leading to a decrease in physical strength, balance, memory, and other cognitive functions. These factors collectively contribute to BADL disability.

### 4.2 CCD is a risk factor for BADL in older adults

The older population commonly experiences deterioration in physical condition, an increased prevalence of chronic diseases, mobility difficulties, and a reduction in daily activities. This study showed that the prevalence of BADL disability in older individuals with CCD was 1.330 times higher than that in those without CCD (13.07% vs. 9.83%, *P* < 0.001). To further investigate the association between CCD and BADL, we conducted PSM analysis on SCD and CCD groups. The analysis revealed a significant difference in the prevalence of BADL disability between the two groups (13.07% vs. 11.39%, 1.179 times, *P* < 0.05). To analyze the possible reasons: first, compared to SCD, CCD has longer courses of disease and complex conditions that would cause long-term suffering (25), thus leading to BADL decline. Second, CCD can lead to multiple uses of drugs, for which compliance is poor. This can cause weakness and obstacles in the management of health (26). Third, the older adults with CCD might consume excessive β receptor blockers and sulfonamides from prescription, which can have side effects including decreased vitality, anorexia, and negative emotions (27). This further causes a decline in quality of life and mobility, leading to BADL disability. In addition, it is worth noting that the older population with CCD often exhibits a lack of health knowledge, which can result in inadequate attention to maintaining a healthy lifestyle. This can contribute to the exacerbation of chronic diseases, the development of complications, and ultimately the decline in BADL.

CCD significantly increases the risk of BADL disability, which is associated with reduced physical exercise and increased negative emotions. In turn, these factors can contribute to the poor prognosis of existing diseases (28, 29). The bidirectional correlation between CCD and BADL disability creates a vicious cycle in the older population. Additionally, it is important to recognize that different combinations of chronic diseases may share common risk factors. In summary, comprehensive measures should be taken to break the vicious circle, including strengthening the dynamic monitoring of chronic disease prevalence, improving community clinic services, and raising awareness among older adults (30). Considering these findings, it is imperative to prioritize the training of general practitioners working in grassroots healthcare institutions. Equipping them with the necessary knowledge and skills is crucial to ensure adequate care for older individuals with BADL disabilities. Furthermore, efforts should be made to disseminate relevant knowledge among the general population, particularly caregivers and family members, to enhance their understanding of the needs and challenges faced by older adults.

### 4.3 Age, educational level, weekly amount of exercise, years of exercise, alcohol intake, social interaction, annual physical examination, retirement benefits, and depression are related to BDAL in older adults

Older individuals with higher levels of education tend to have a stronger awareness of disease prevention and healthcare. Educational level is also related to social status and economic resources. These factors can contribute to better health outcomes and wellbeing (31). Furthermore, this study revealed a positive correlation between engagement in regular exercise and BADL levels. Exercise can help maintain muscle mass, improve cardiovascular function, and regulate mental wellbeing, all of which contribute to improving BADL (32). It is important to note that alcohol intake has detrimental effects on the digestive and neurological systems. Excessive alcohol intake can increase the burden on the liver and kidneys, potentially leading to BADL decline (33). High participation in social interaction can help avoid negative emotions and improve neurological function (34), which might help maintain BADL. The annual physical examination can help to discover changes in health status and to take preventive measures promptly, which is beneficial to BADL maintenance. Moreover, adequate retirement pension allows older individuals to allocate relatively sufficient resources to quality of life and medical services (35). Finally, this study found that depression was a risk factor for BADL, consistent with a previous study (36). A possible reason might be that depression could cause brain damage and cognitive impairment, which would further lead to BADL decline.

### 4.4 Suggestions and outlook

In practical terms, this study provides valuable insights into fostering collaboration among individuals, family members, society, and healthcare institutions. First, it is recommended that older individuals cultivate a healthy lifestyle by engaging in regular physical activity and minimizing alcohol intake. Second, family members should prioritize active communication with older adults, including regular check-ups, attention to their mental wellbeing, and the provision of social support. Third, from a societal standpoint, it is crucial to strengthen welfare benefits for older adults. Diverse activities that enrich the lives of older individuals should be implemented to prevent depression and alleviate feelings of isolation. Finally, healthcare institutions should publicize the significance of the prevention and treatment of chronic diseases through primary care, actively carry out disease screening, and facilitate early detection and intervention to safeguard BADL.

By collaboratively addressing these issues, strong support can be provided to enhance the BADL of the older population. This approach not only helps improve the quality of life of older adults but also alleviates the burden on families and society.

## 5 Strengths and weaknesses of the study

In our study, we observed a progressive relationship between no CCD, SCD, and CCD. PSM was used to eliminate the confounding factors and enhance the reliability of the results. However, this study had several weaknesses. The cross-sectional design used in this study makes it difficult to infer causality. Therefore, bidirectional causality cannot be ruled out. Considering this, future research endeavors are planned to adopt tracking designs and randomized interventions to further explicate the causal relationships between variables. Second, it is worth mentioning that all samples included in this study were drawn from Zhengzhou. Caution must be exercised when attempting to generalize the findings to other populations. To address this issue, future research endeavors should aim to expand the coverage of survey objects and collect data from a wider range of provinces.

## 6 Conclusion

The prevalence of BADL disability in the older adults with CCD is significantly higher than that of the older adults with SCD and no CCD. CCD, age, educational level, weekly amount of exercise, years of exercise, alcohol intake, social interaction, annual physical examination, retirement benefits, and depression are the factors associated with BADL.

## Data availability statement

The raw data supporting the conclusions of this article will be made available by the authors, without undue reservation.

## Ethics statement

The studies involving humans were approved by Ethics Committee of Zhengzhou University (Ethical number: ZZUIRB2022-07). The studies were conducted in accordance with the local legislation and institutional requirements. Written informed consent for participation was not required from the participants or the participants' legal guardians/next of kin in accordance with the national legislation and institutional requirements.

## Author contributions

HZ: Conceptualization, Data curation, Formal analysis, Methodology, Project administration, Software, Supervision, Visualization, Writing – original draft, Writing – review & editing. CM: Writing – review & editing. RW: Visualization, Writing – review & editing. WZ: Software, Visualization, Writing – review & editing. WW: Visualization, Writing – review & editing. YL: Writing – review & editing. SW: Writing – review & editing. AL: Writing – review & editing. HJ: Writing – review & editing. GL: Writing – review & editing. JZ: Writing – review & editing. XC: Writing – review & editing. QT: Conceptualization, Funding acquisition, Investigation, Project administration, Resources, Supervision, Validation, Writing – review & editing.
