# Supplementary material for: Influence of comorbidity of chronic diseases on basic activities of daily living among older adults in China: a propensity score-matched study
Source: Front Public Health. 2024 Apr 4;12:1292289. doi: 10.3389/fpubh.2024.1292289 (PMC11024351; doi:10.3389/fpubh.2024.1292289)

Appendix A Modified Barthel Index scale

| BADL Item | Complete independence | Basic independence | Requirement of assistance | Requirement of a lot of assistance | Complete dependence |
| --- | --- | --- | --- | --- | --- |
| Fecal continence | 10 | 8 | 5 | 2 | 0 |
| Micturition continence | 10 | 8 | 5 | 2 | 0 |
| Eating | 10 | 8 | 5 | 2 | 0 |
| Dressing | 10 | 8 | 5 | 2 | 0 |
| Bathing | 10 | 8 | 5 | 2 | 0 |
| Personal hygiene | 10 | 8 | 5 | 2 | 0 |
| Toileting | 10 | 8 | 5 | 2 | 0 |
| Moving | 10 | 8 | 5 | 2 | 0 |
| Walking | 10 | 8 | 5 | 2 | 0 |
| Climbing stairs | 10 | 8 | 5 | 2 | 0 |

Appendix B Assignment of variable

| **Variable** | **Assignment** |
| --- | --- |
| Explained variable |  |
| BADL | BADL no disability =1，BADL disability = 0 |
| Explanatory variable |  |
| Condition of CCD and no CCD | No CCD =0，CCD =1 |
| Condition of SCD and CCD | SCD =0, CCD =1 |
| Control variables |  |
| Sociodemographic characteristics |  |
| Gender | Female =0，Male =1 |
| Type of residence | Urban =1，Rural =0 |
| Ethnicity | Han =1，minority nationality =0 |
| Educational level | Junior high school or above =0；  Under junior high school =1 |
| BMI | 18.5 kg/m^2^~23.9 kg/m^2^=1  Else =0 |
| Social support |  |
| Cohabitation | Living with others =1，Living Alone =0 |
| Daily care from offspring | Yes =1，No =0 |
| Social interaction | Yes =1，No =0 |
| Health behavior |  |
| Smoking | No =0，Yes =1 |
| Alcohol intake | No =0，Yes =1 |
| Annual physical examination | Yes =1，No =0 |
| Balanced diet | Yes =1，No =0 |
| Economic status |  |
| Retirement benefits | Yes =1，No =0 |
| Mental health |  |
| Depression | Not Suffering =1，Suffering =0 |

APPENDIX C. Comparison of the general information of the group with CCD and no CCD before and after PSM


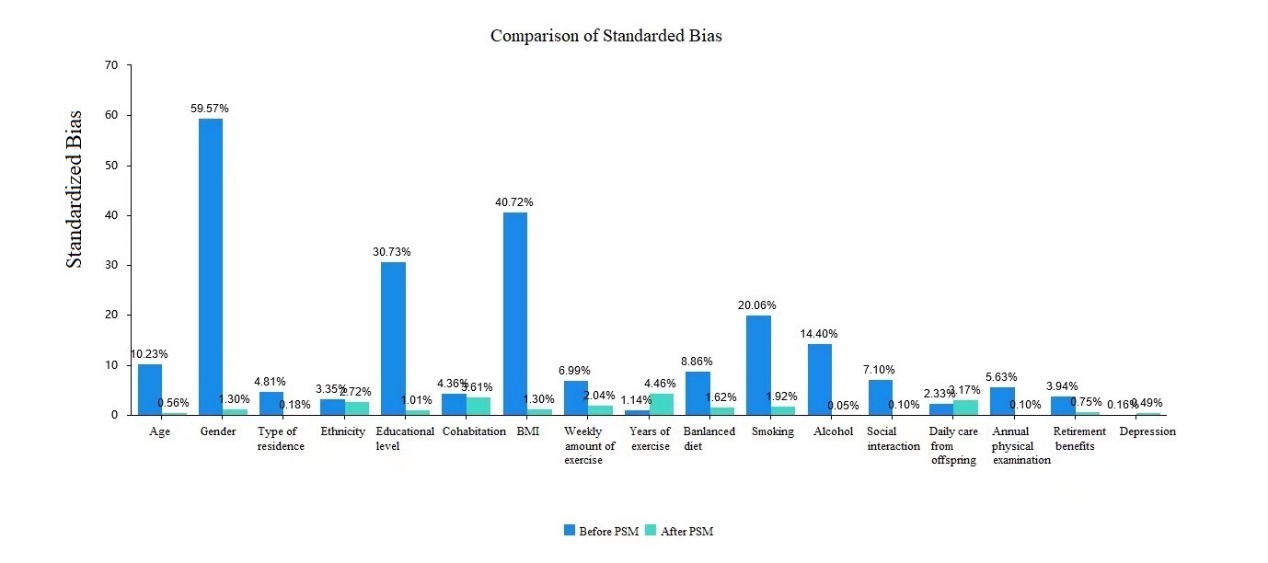


APPENDIX D. Comparison of the general information of the group with CCD and with SCD before and after PSM


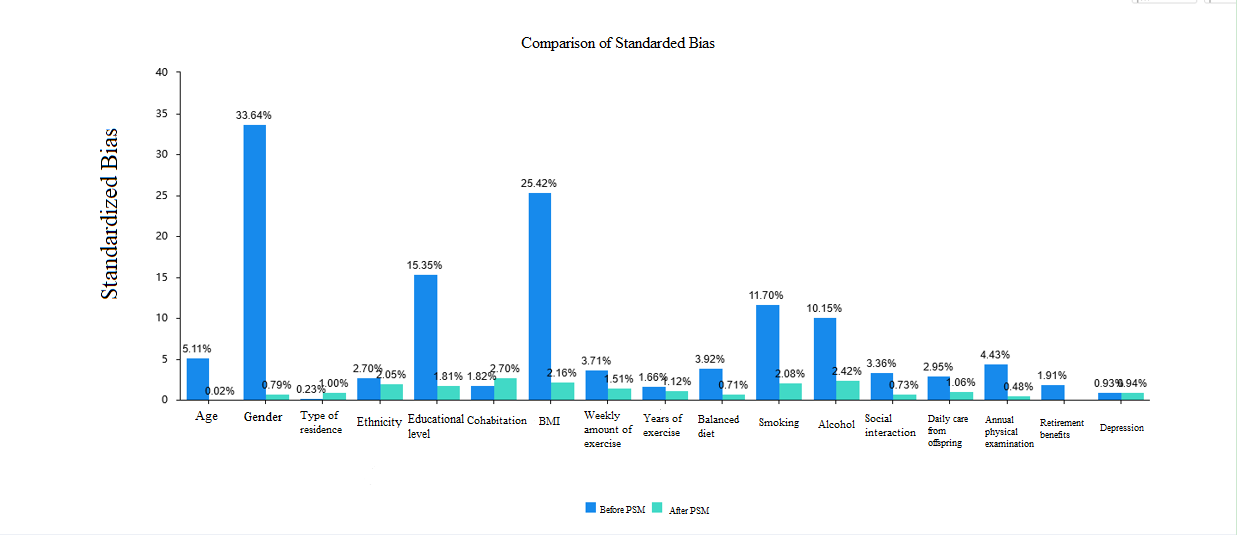

Supplement: Supplementary file 1 [file Data_Sheet_1.DOCX]
